# Computational survey of humin formation from 5-(hydroxymethyl)furfural under basic conditions†

**DOI:** 10.1039/d3ra02870d

**Published:** 2023-05-31

**Authors:** Keisuke Tashiro, Masato Kobayashi, Kiyotaka Nakajima, Tetsuya Taketsugu

**Affiliations:** a Graduate School of Chemical Sciences and Engineering, Hokkaido University Sapporo 060-0810 Japan; b Department of Chemistry, Faculty of Science, Hokkaido University Sapporo 060-0810 Japan k-masato@sci.hokudai.ac.jp; c WPI-ICReDD, Hokkaido University Sapporo 001-0021 Japan; d ESICB, Kyoto University Kyoto 615-8520 Japan; e Institute for Catalysis, Hokkaido University Sapporo 001-0021 Japan

## Abstract

A comprehensive reaction-path search for the oligomerization of 5-(hydroxymethyl)furfural (HMF) based on quantum chemical calculations was conducted to clarify the mechanism of humin formation in the oxidation of HMF to furan-2,5-dicarboxylic acid (FDCA), in which humin is a typical macromolecular byproduct. The present procedure repeatedly utilizes the multi-component artificial-force-induced reaction (MC-AFIR) method to investigate multistep oligomerization reactions. Although humin formation has been reported even in reagent-grade HMFs with 97–99% purity during their storage at low temperatures, no direct addition path of two HMFs with <185 kJ mol^−1^ barrier has been found, suggesting humin formation is caused by a reaction with impurities. Based on the reaction conditions, we considered the reactions of HMF + H_2_O, HMF + OH^−^, and HMF + O_2_ and identified three reaction paths with <65 kJ mol^−1^ barrier for the reaction of HMF + OH^−^. Further, the suppression of humin formation by the acetal protection of HMF is computationally confirmed.

## Abstract 2

By repeatedly utilizing the MC-AFIR method, a comprehensive reaction-path search for the oligomerization of HMF was conducted to clarify the mechanism of humin formation in the oxidation of HMF to FDCA.

## Graphical abstract

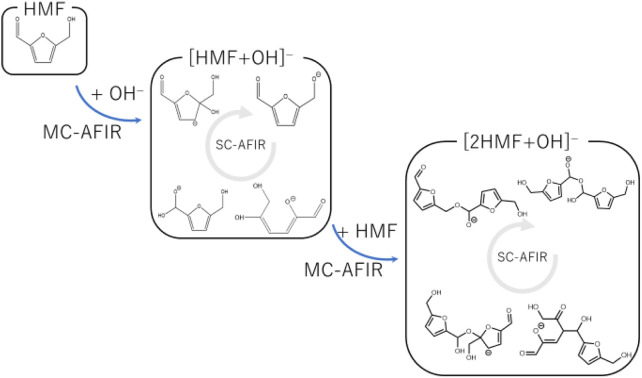

## 1 Introduction

With limited resources, breaking free from dependence on fossil-derived chemicals is a major goal of sustainable development. Inedible biomass resources are recognized as alternatives to fossil fuels.^1^ FDCA^2^ is a key molecule in biomass chemistry because it can be used as an inedible biomass feedstock for polyethylene terephthalate (PET) substitutes. The polymerization of FDCA yields polyethylene furanoate, which has potential to replace fossil-derived PET and terephthalate-based resins due to better gas barrier properties than PET. In particular, the oxidation of HMF to FDCA has attracted considerable attention and has been enthusiastically studied worldwide.^3–6^ The reaction is renowned as a system that produces an undesirable solid byproduct called humin because of the complex reaction network. The humin formation reduces the production efficiency of FDCA, thereby preventing the large-scale production of FDCA in industry. For instance, humin production steeply increases from a 4% yield with a 1 wt% HMF solution to a 47% yield with a 10 wt% HMF solution.^7^ Understanding the reaction mechanism to produce humin, based on quantum chemical calculations, is critical for the rational design of conditions (*e.g.*, catalysts, temperature, and solvent) for efficient FDCA production; however, it has not been extensively studied. A major factor hindering such theoretical work is the lack of methodologies that can systematically analyze perplexing reaction networks.

The humin formation from HMF has been experimentally studied. Horvat *et al.* propose that under acidic conditions, acid-catalyzed hydrolysis leads to a furan ring-opening reaction to form humin.^8,9^ Patil and Lund demonstrate that polymerization to form humin occurs by the aldol addition of the ring-opened product (2,5-dioxo-6-hydroxy-hexanal) with HMF.^10^ The structure of humin has been analyzed by one-dimensional (1D) and two-dimensional (2D) solid-state nuclear magnetic resonance (NMR) measurements.^11,12^ Recently, biomass processing using ionic liquid solvents has attracted significant attention.^13^ HMF is degraded to humins even in ionic liquid solvents, and its structure has been revealed by analytical techniques, such as Fourier-transform infrared (FT-IR) spectroscopy, ^13^C-NMR, and X-ray photoelectron spectroscopy (XPS).^14^ Furthermore, it has been reported that humin is formed even in pure HMF during its storage at low temperatures.^15^ Nakajima *et al.* have reported that HMF–acetal, which is produced by the reaction of HMF with 1,3-propanediol (PD), exhibits significantly higher thermal stability against thermal degradation than HMF.^7^ The oxidation and oxidative esterification of HMF–acetal affords FDCA and its esters in excellent yield and selectivity, even in concentrated solutions (>10 wt%).^16^ The high stability of HMF–acetal is derived from full protection of highly reactive formyl group that most likely induces humin formation. Na_2_CO_3_ used in these oxidation reactions is essential for stabilizing the acetal moiety against undesirable hydrolysis and acting as a neutralizing salt to dissolve FDCA in water or methanol. Further clarification of the humin formation mechanism based on quantum chemical calculations is desirable for the effective use of HMF in a variety of reactions.

In this study, we conduct a comprehensive reaction-path search for humin formation under the conditions of FDCA formation by the oxidation of HMF using the artificial-force induced reaction (AFIR) method, which is a part of the global reaction route map (GRRM) strategy developed by Maeda *et al.*^17–20^ The multi-component (MC) AFIR method is applied to candidate bimolecular reactions in the oligomerization of HMF, which enables the systematic exploration of association reactions. Considering the basic reaction conditions, the following reactions are identified as candidates for the initial elementary reactions: HMF + HMF, HMF + H_2_O, HMF + OH^−^, and HMF + O_2_. By repeatedly applying the MC-AFIR method to the reaction of HMF with the newly obtained chemical species, the formation process of HMF oligomers can be obtained. The results show that humin formation is unlikely to occur when only HMF and H_2_O are present in the reaction system: the barrier for the reaction between HMF and HMF or HMF and H_2_O is at least 186.5 kJ mol^−1^. In contrast, the reaction between HMF and OH^−^ yields many intermediates with a low barrier. Thus far, we have obtained a trimer of HMF in our simulations under basic conditions. We conclude that these reactions are the initial steps in the formation of humin from HMF under basic conditions. Furthermore, the mechanism of the suppression of humin formation in concentrated solutions by acetal protection is computationally investigated.

## 2 Procedure and computational details

The Gaussian 16 software package^21^ was employed for the quantum chemical calculations. All structures were optimized using the Kohn–Sham density functional theory with the B3LYP functional^22,23^ with Grimme's D3 dispersion correction (without Becke–Johnson damping).^24^ The 6-31+G(d) basis set^25–28^ was used for all atoms. Water solvent effects were considered using the integral equation formalism variant of the polarizable continuum model (IEFPCM).^29^

**Fig. 1:**
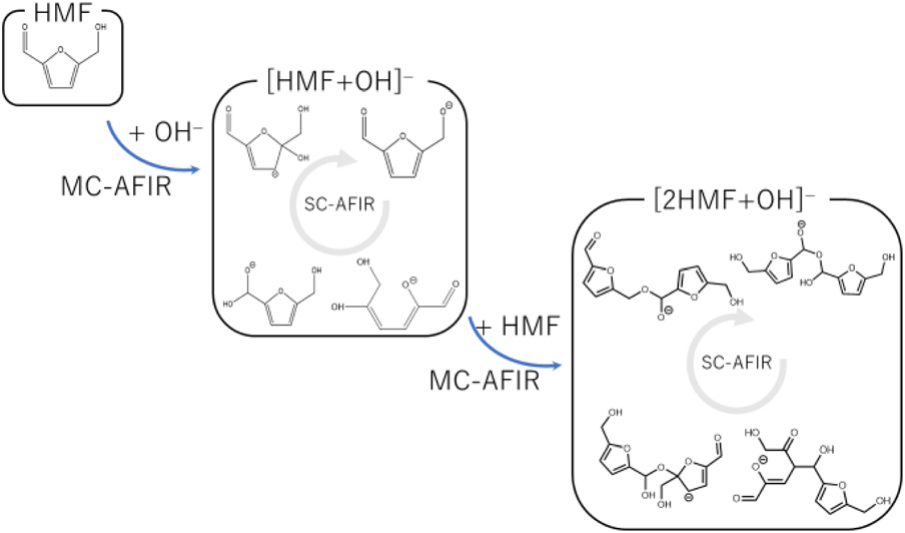
Reaction search scheme using multi-step MC-AFIR method.

To systematically search for reaction paths, we employed the AFIR method implemented in the GRRM17 program,^19^ which induced a reaction by applying an artificial force between two moieties. The following procedure was employed to analyze the multistep polymerization reactions because the AFIR method implemented in GRRM17 cannot directly handle multistep reactions with different participating molecules (Fig. 1). First, the reactions listed in Table 1 were selected as candidates for the initiation reactions in a basic aqueous solution. The intermediates derived from these reactions were obtained using the MC-AFIR method.^16^ Thereafter, the reactions of these intermediates with HMF were selected as candidates for new bimolecular reactions and examined using the MC-AFIR method. For some intermediates, such as ring-opening systems, further single-component (SC)-AFIR calculations were performed to identify intramolecular rearrangements and conformational changes. By repeating these steps, we could comprehensively investigate the process from the initial humin formation to the HMF trimer. Parameter *γ*, which represents the magnitude of the artificial force used in the AFIR method, was set in the range of 200–500 kJ mol^−1^. The number of samples was set to increase as the size of the system increased or as the system was considered more important. Specifically, the number of samples was set to 100–500 for the initial reaction, 300–600 for the dimerization reaction, and 500–2000 for the trimerization reaction. The reactions obtained by the MC-AFIR method were clustered into elementary chemical reactions, *i.e.*, if the reactants and products of the two reactions were the same in the canonical simplified molecular-input line-entry system (SMILES) representation, these reactions were considered to be chemically identical. The conversion of molecular geometry into canonical SMILES was performed using the Open Babel program.^30,31^

**Table 1:** Candidates of the initial reactions for which the reaction-path searches were conducted

Candidate	Lowest activation energy/kJ mol^−1^
HMF + HMF	186.5
HMF + H_2_O	209.9
HMF + OH^−^	30.7
HMF + O_2_ (singlet)	111.6
HMF + O_2_ (triplet)	135.7

## 3 Results and discussion

**Fig. 2:**
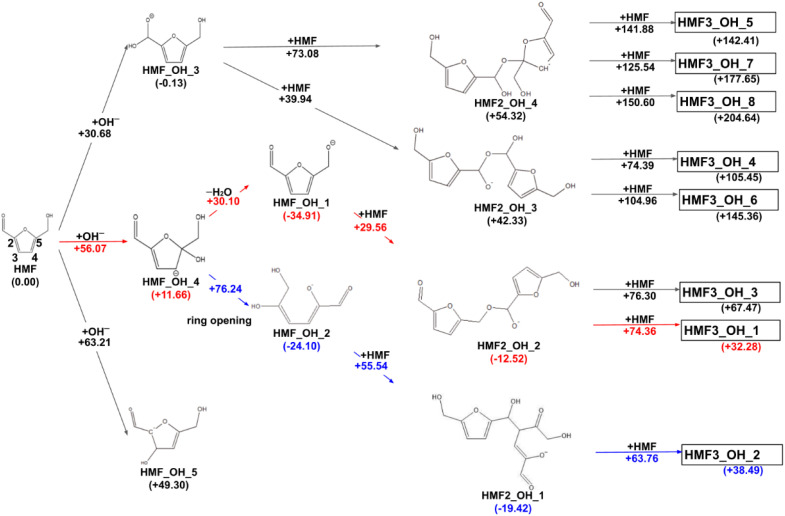
Reaction route map for the initial steps of humin formation under basic conditions; red arrows show the pathway leading to the most stable trimer, and blue arrows show the pathway leading to the most stable dimer. All structure xyz data are listed on GitHub (https://github.com/k-masato/HMF_geometries).

**Fig. 3:**
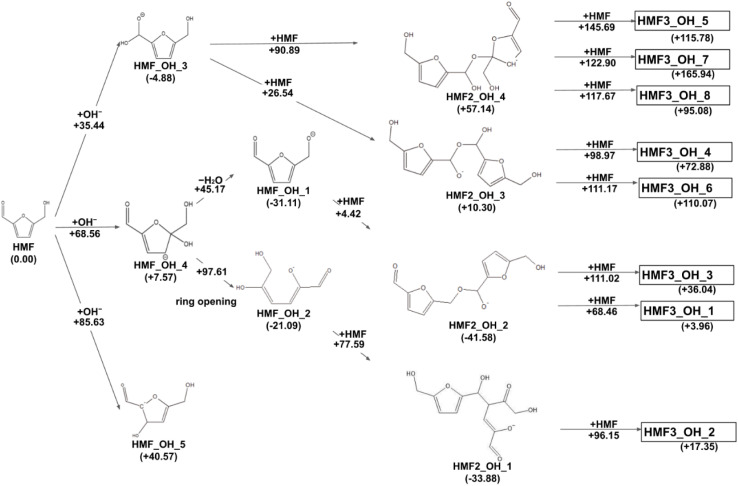
Reaction route map for the initial steps of humin formation recalculated with ωB97X-D/aug-cc-pVTZ level of theory at the geometries optimized by B3LYP-D3/6-31+G(d).

The reaction paths obtained from the series of AFIR calculations are summarized in Fig. 2. The values in parentheses next to the labels are Gibbs energies (in kJ mol^−1^) relative to the reactants. Each arrow represents an elementary reaction, and the value above it is the Gibbs activation energy (in kJ mol^−1^). The following subsections provide detailed discussions of each process. We also calibrated the electronic energy by single-point calculations with the ωB97X-D^32^/aug-cc-pVTZ^33^ level of theory. The obtained Gibbs energies are shown in Fig. 3. While the energy values are different to some extent, the following discussion remains qualitatively the same for the values in both levels of theory. For consistency, when referring to specific energy values, we use the values shown in Fig. 2.

### 3.1. Initiation reactions of humin production

**Fig. 4:**
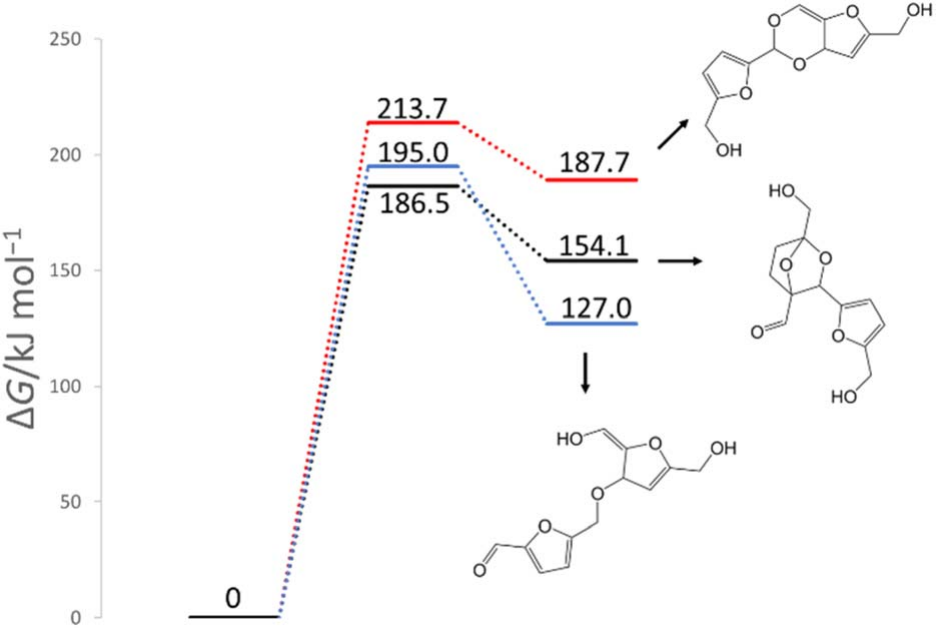
Gibbs energy diagram for the reactions of HMF + HMF under neutral conditions (298 K, 1 atm).

In previous experimental reports, HMF of 97–99% purity was degraded to a dimer in approximately two weeks, even at room temperature.^15^ At 473 K, the degradation was almost complete in 2 h.^7^ First, the reactions of the two HMF molecules were explored using the MC-AFIR method, and a total of 79 transition states (TSs) were located, including three elementary bimolecular addition reactions. The energy diagrams of these reactions are shown in Fig. 4. The bimolecular reaction with the lowest activation energy was a Diels–Alder type reaction with an activation energy of 186.5 kJ mol^−1^, which is considerably high to proceed at room temperature. Although the effect of the water solvent was considered in the present calculations, the results did not change when the solvent was varied to HMF, for which parameters *ε* (dielectric constant) and *R*_solv_ (solvent radius) were determined as *ε* = 26.38 and *R*_solv_ = 3.384 Å, based on molecular dynamics simulations. Therefore, the initial degradation of HMF proceeds *via* a reaction with impurities. Among the initial reactions of the HMF monomers listed in Table 1, the reaction with OH^−^ had the lowest activation energy of 30.7 kJ mol^−1^, while the reactions with H_2_O and O_2_ (triplet) had those of 209.9 and 135.7 kJ mol^−1^, respectively (Gibbs energy diagrams for the reactions with H_2_O and O_2_ are shown in Fig. S1 and S2,† respectively, in the ESI†). The MC-AFIR calculations for the reaction of HMF + OH^−^ found 30 TSs. For the chemical reactions with activation energies below 100 kJ mol^−1^, we observed three hydroxylation reactions of the carbons at positions 3 and 5 of the furan ring and the formyl carbon, affording HMF_OH_5, HMF_OH_4, and HMF_OH_3, respectively (Fig. 2). This is consistent with chemical intuition because a negative charge on the oxygen atom of the formyl group would draw a resonance structure with a positive charge on one of these three carbons. Further, a reaction involving the carbon at position 4 was found in the MC-AFIR calculation; however, its activation energy was 121.6 kJ mol^−1^, which is considerably higher than the above energy and is not shown in Fig. 2. Among these three reactions, the formation of HMF_OH_3 was found to be favorable regarding activation energy. Notably, HMF_OH_3 is the anionic form of the 5-hydroxymethyl-2-furancarboxylic acid that produces FDCA. However, HMF_OH_4 induced ring-opening and dehydration reactions, producing more stable HMF_OH_2 and HMF_OH_1, respectively, with relatively small activation energies. Consequently, the reaction of HMF with OH^−^ provided five intermediates (*i.e.*, HMF_OH_*X*, *X* = 1–5) with activation energies below 100 kJ mol^−1^. The labels “HMF_OH_*X*” are numbered based on the stable species, *i.e.*, HMF_OH_1 produced by the dehydration of HMF_OH_4 is the most stable.

### 3.2. Comparison with the reactions of PD-HMF

**Fig. 5:**
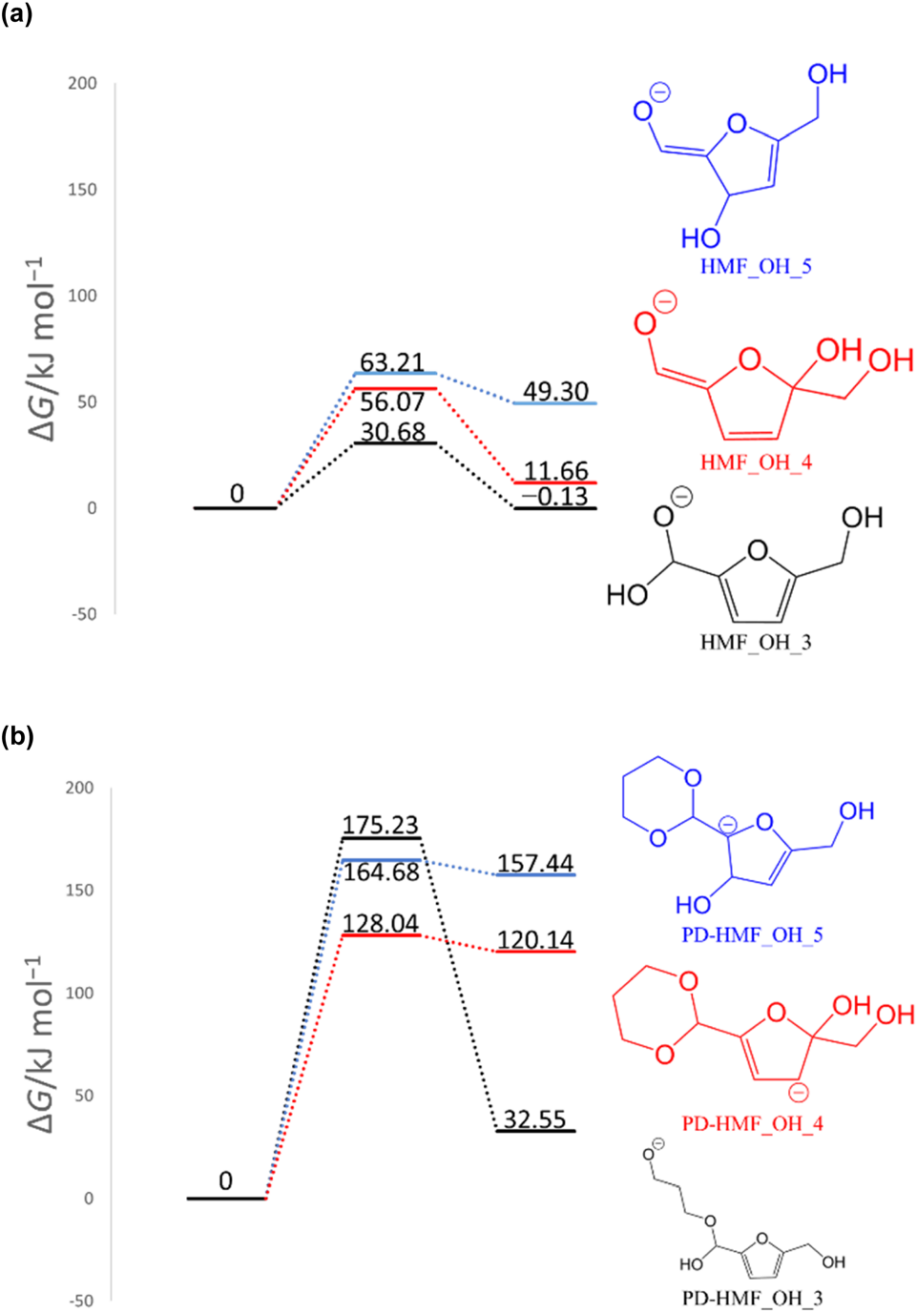
Gibbs energy diagrams of the reactions of (a) HMF + OH^−^ and (b) PD-HMF + OH^−^ (298 K, 1 atm).

**Fig. 6:**
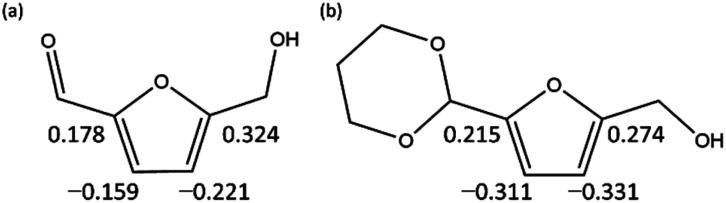
Natural electron populations on furan rings of (a) HMF and (b) PD-HMF.

To computationally verify the effect of acetal protection, the reaction of PD-HMF (HMF–acetal protected by PD) with OH^−^ was examined. Fig. 5 shows the hydroxylation paths at carbons 3 and 5 of the furan ring of PD-HMF and the protected formyl carbon, corresponding to PD-HMF_OH_5, PD-HMF_OH_4, and PD-HMF_OH_3 formation, respectively. This protection directly inhibits hydroxylation at the formyl carbon, resulting in an activation energy of 175.2 kJ mol^−1^ to obtain PD-HMF_OH_3, approximately 145 kJ mol^−1^ higher than the activation energy to obtain HMF_OH_3. Interestingly, acetal protection increased the activation energy to obtain PD-HMF_OH_4 and PD-HMF_OH_5 by approximately 70 and 100 kJ mol^−1^, respectively. This is mainly due to the loss of π-conjugation with formyl C=O, which destabilizes the products. Further, this can be interpreted in terms of electrophilicity. Fig. 6 shows the results of the natural population analysis^34,35^ for HMF and PD-HMF. The computational level was B3LYP-D3/6-31+G(d), with the same IEFPCM solvation model as the energy calculation. Acetal protection reduced the charge on the carbons at position 3/5 of the furan ring from −0.159/0.324 to −0.311/0.274, suggesting that the reaction with OH^−^ at these positions was inhibited. Thus, the stabilization of HMF by acetal protection was theoretically proven.

### 3.3. Dimerization and trimerization of HMF

The dimerization reaction proceeded for HMF_OH_1, HMF_OH_2, and HMF_OH_3. The dimerization reaction for HMF_OH_5 is not considered in this study because the minimum barrier height is 196.2 kJ mol^−1^. The most stable dimer (HMF2_OH_1) was obtained by an aldol-like reaction of HMF_OH_2 with HMF, with a barrier height of 55.5 kJ mol^−1^. The dimerization reaction with the lowest barrier was HMF_OH_1 + HMF, where O^−^ attacked the formyl group of the new HMF to yield HMF2_OH_2 with a barrier of 29.6 kJ mol^−1^. The trend toward the lowest activation energy for the dimerization reaction at the formyl carbon was similar to that observed for the reaction at HMF + OH^−^. These dimers have negative relative energies and may be important intermediates contributing to humin formation. For HMF_OH_3, there are two dimerization pathways leading to HMF2_OH_3 and HMF2_OH_4, whose relative energies were considerably higher than those of the other dimers. HMF_OH_3 is a derivative of 5-hydroxymethyl-2-furan carboxylic acid (HMFCA), an intermediate in FDCA synthesis. This result suggests that the formation of HMF_OH_3 (or HMFCA) does not lead to humin formation.

**Fig. 7:**
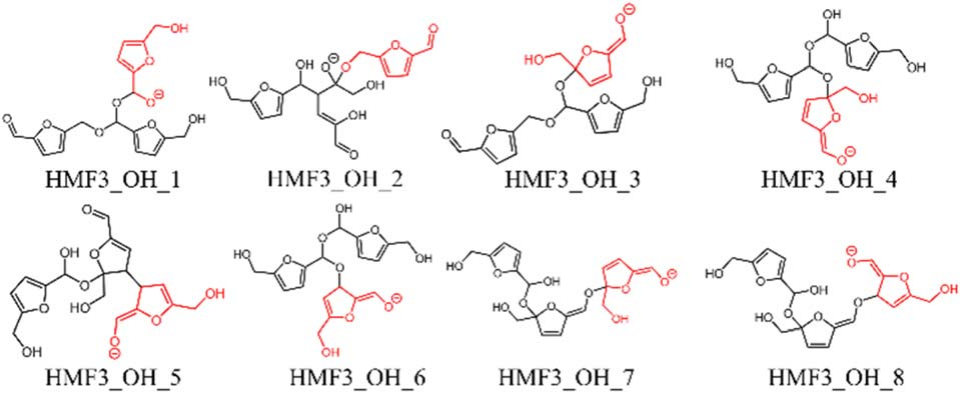
Trimer structures obtained in this study; the last attacking HMF is shown in red.

In this study, eight pathways for trimer formation were identified based on the chemical structures shown in Fig. 7. The reaction barriers for trimer formation were all higher than 60 kJ mol^−1^, and the positive relative energies of all trimers suggest that trimer formation is slower than dimer formation under basic conditions.

### 3.4. Discussion on the FDCA formation from HMF and PD-HMF under basic condition

In the reaction that produces FDCA from HMF, the oxidation of the aldehyde corresponds to the initial reaction. The activation energy for the formation of HMF_OH_3 was the lowest among the three initial pathways. The byproducts of the hydroxylation reaction, particularly HMF_OH_4 and the subsequent HMF_OH_1 and HMF_OH_2, may be involved in humin formation, a side reaction of FDCA formation. Additionally, the activation energy to produce HMF_OH_4 was less than 60 kJ mol^−1^, which is lower enough as the reaction proceeds at room temperature. This is consistent with the experimental fact that the side reaction proceeds more likely as the HMF concentration increases. Conversely, no OH^−^ was involved in the initial oxidation step of PD-HMF because it is an alcohol oxidation of –CH_2_OH. In other words, in the reaction using PD-HMF, all reactions with OH^−^ observed for HMF were suppressed by acetal protection. The initial reaction was guided by the oxidation of alcohol, and humin formation was avoided.

Although we did not consider the catalyst or molecular oxygen on the metal support in this study, the results shown in this paper suggest a deeper understanding of humin formation in various basic solutions and the effect of acetal protection on HMF.^36–38^

## 6 Conclusion

In this study, we performed systematic reaction-path-search calculations for the formation of humin from HMF under basic conditions. Although humin formation has been reported even with pure HMF, the direct addition reaction path (HMF + HMF) has a barrier of at least 186.5 kJ mol^−1^ and is unlikely to occur at room temperature. In contrast, for the HMF + OH^−^ reaction, three pathways with barriers of less than 65 kJ mol^−1^ were identified. HMF_OH_4 was found to be an important intermediate that produces HMF dimers with a low reaction barrier. However, trimerization was found to have a higher reaction barrier than dimerization. Further, AFIR calculations were performed for the system in which HMF was replaced with PD-HMF to verify the change in reactivity due to acetal protection. When acetal-protected PD-HMF was used instead of HMF, the activation energies of the reaction involving the protected formyl carbon and the other two initial reactions significantly increase. This result explains the experimental stability of the PD-HMF under basic conditions and reinforces the validity of the initial reaction for humin formation obtained in this study. From the aspect of computational chemistry, this study demonstrates the validity of the multistep application of the MC-AFIR method for unidentified multistep reactions.

## Conflicts of interest

There are no conflicts to declare.

## Supplementary Material

RA-013-D3RA02870D-s001
