# Supplementary material for: Computational survey of humin formation from 5-(hydroxymethyl)furfural under basic conditions
Source: RSC Adv. 2023 May 31;13(24):16293–9. doi: 10.1039/d3ra02870d (PMC10230611; doi:10.1039/d3ra02870d)
Supplement: RA-013-D3RA02870D-s001 [file RA-013-D3RA02870D-s001.pdf]

Electronic Supplementary Material (ESI) for RSC Advances.
This journal is © The Royal Society of Chemistry 2023

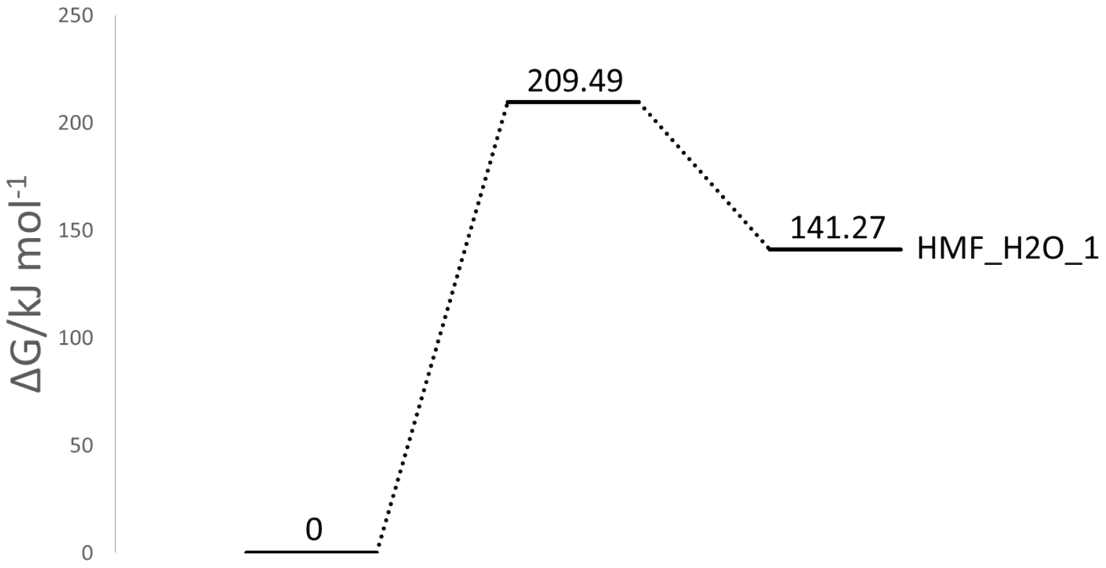

Fig. S1 Gibbs energy diagram or the reaction of HMF + H_2O under neutral conditions (298 K, 1 atm).

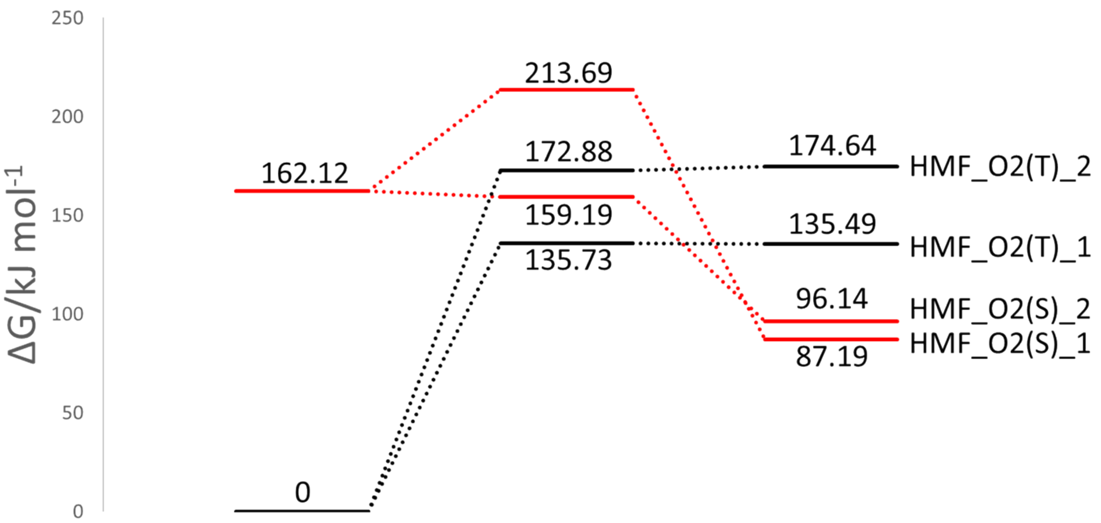

Fig. S2 Gibbs energy diagram for the reactions of HMF + O_2 under neutral conditions (298 K, 1 atm).

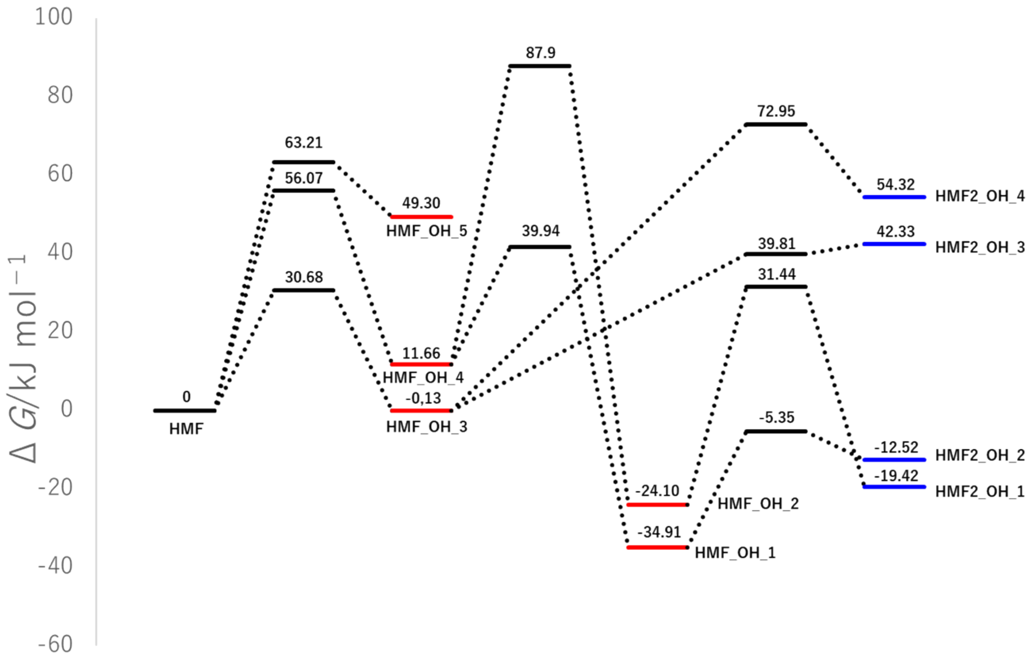

Fig. S3 Gibbs energy diagram for Fig. 2 up to dimer formation. Red and blue lines indicate the energies of $[HMF + OH]^{-}$ and $[2HMF + OH]^{-}$, respectively.

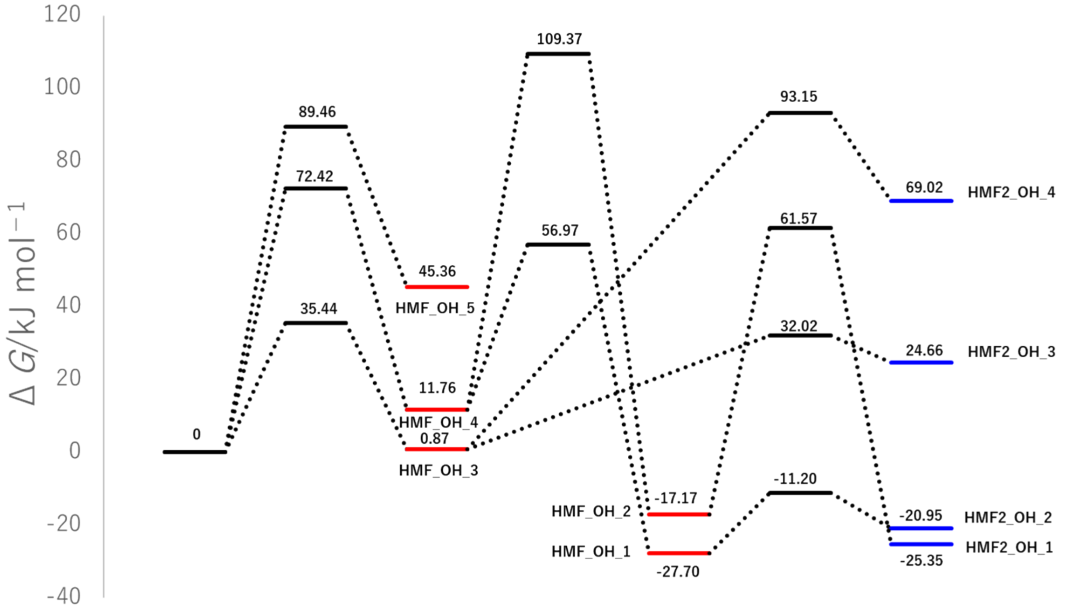

Fig. S4 Gibbs energy diagram for Fig. S1 up to dimer formation. Red and blue lines indicate the energies of [HMF + OH]$^{-}$ and [2HMF + OH]$^{-}$, respectively.
